# Oral Metronidazole Desensitization for Immunoglobulin E (IgE)-Mediated Hypersensitivity

**DOI:** 10.7759/cureus.26849

**Published:** 2022-07-14

**Authors:** Chantae C Hollis, Chamunorwa Mlauzi, Michael Ashton

**Affiliations:** 1 Internal Medicine, Bermuda Hospitals Board, Paget, BMU; 2 Medicine, John F. Kennedy University School of Medicine, Willemstad, CUW; 3 Pharmacy, Bermuda Hospitals Board, Paget, BMU; 4 Infection & Diseases, Bermuda Hospitals Board, Paget, BMU

**Keywords:** desensitization, metronidazole, allergic hypersensitivity, sexually transmitted infection (sti), trichomoniasis

## Abstract

Low efficacy has been reported for the treatment of *Trichomonas vaginalis* with antimicrobial agents other than the 5-nitroimidazole class. Patients with immunoglobulin E (IgE)-mediated hypersensitivity reactions to drugs in this class, including metronidazole and tinidazole, may benefit from a desensitization treatment. We present two patients with past medical histories of IgE-mediated hypersensitivity reactions to metronidazole who were successfully treated with metronidazole using a modified protocol. The first patient was a 39-year-old female diagnosed with trichomoniasis who had a previous reaction to metronidazole characterized by urticaria and pruritus. The second patient was a 53-year-old female who had a history of untreated trichomoniasis due to a previous anaphylactic reaction to metronidazole. Both patients were successfully treated in the intensive care unit (ICU) without signs and symptoms of the reaction. Physicians may use a desensitization protocol for patients with a presumed IgE-mediated hypersensitivity when only the 5-nitroimidazole class is efficacious for the treatment of trichomoniasis.

## Introduction

Metronidazole or tinidazole of the 5-nitroimidazole class of drugs are presently the only pharmacologic agents for the curative therapy of trichomoniasis [1]. *Trichomonas vaginalis* is a flagellated protozoan parasite that is the etiologic cause of trichomoniasis, the most commonly curable, non-viral sexually transmitted infection (STI) in the world [2-4]. It is estimated that trichomoniasis has an annual incidence of 276 million cases and a prevalence of 187 million infected individuals [4]. Women with trichomoniasis may be asymptomatic or have a range of symptoms including mild vaginal itching, foul-smelling yellow or greenish vaginal discharge, to severe inflammation and pain, while men are frequently asymptomatic and may experience non-gonococcal urethritis [5,6]. Without curative treatment, the infection can become chronic, increasing the risk of HIV and other sexually transmitted infections as well as adverse pregnancy outcomes [3]. Currently, two dosing regimens have been recommended: either 500 mg of metronidazole or tinidazole twice a day for seven days or a 2 g single dose of metronidazole [1,4]. Although other pharmacologic agents, including clotrimazole and combination sulfanilamide, aminacrine hydrochloride, and allantoin suppositories, have been cited in the literature, oral metronidazole is the most efficacious treatment for *T. vaginalis* [7]. In the case of patients with an immunoglobulin E (IgE)-mediated allergy to metronidazole or tinidazole, it is suggested for patients to be desensitized as opposed to using alternative pharmacologic agents, which have been shown to be effective in women with 5-nitroimidazole hypersensitivity [1,5,8,9].

Drug desensitization is a procedure that allows temporary tolerance of pharmacologic agents in patients with a known or suspected hypersensitivity reaction [10], therefore, allowing patients to safely receive a drug continuously for the recommended time of treatment [11]. IgE-mediated hypersensitivity reactions can range from mild pruritus, vomiting, nasal congestion, pharyngeal pruritus, tingling of the lips, and urticaria, to more life-threatening systemic anaphylactic reactions requiring hospitalization and intubation [8,12]. Importantly, the tolerance is temporary; once the patient stops taking the drug and it is completely eliminated from the circulation, the hypersensitivity will return [11]. Therefore, maintaining a state of desensitization would require the continuous presence of the drug in the patient’s body through regular dosing, with no missed doses [11].

To our knowledge, two metronidazole desensitization protocols exist in the literature [5,8]. For the desensitization of both patients presented in our case report, the clinical team elected to follow the modified protocol published by Gendelman et al. [5], which resulted in the formulation of a precise, metronidazole desensitization dilution protocol.

## Case presentation

Case 1

A 39-year-old black female gravida 6, para 4, abortus 2, presented to the local health department with the complaint of mild intermittent yellow vaginal discharge for several days and white vaginal discharge upon voiding. Initial results from a gram stain and wet prep were negative for *Trichomonas vaginalis* and negative for yeast, but positive for bacterial vaginosis. Further laboratory investigations revealed a positive *Trichomonas* antigen test. The patient had a history of recurring bacterial vaginosis, chlamydia, gonorrhea, and trichomoniasis several years ago. The patient reported several medication allergies, including aspirin, diclofenac, and metronidazole, in which she developed a presumed IgE-mediated hypersensitivity characterized by urticaria and generalized pruritus. The patient was referred to the infectious diseases' specialist for consultation on metronidazole desensitization.

On physical exam, the patient was noted to be afebrile. Vital signs included a blood pressure of 142/83 and a heart rate of 69 beats per minute. The patient was educated on her diagnosis and it was explained that it would be best practice to treat trichomoniasis due to the risk of disease progression. The patient was counseled on the benefits and risks of the procedure, and informed consent was obtained by the consultant. She received two grams of gradual escalation treatment with metronidazole in the intensive care unit (ICU) with continuous monitoring.

The patient was pre-treated intravenously with 20 mg of dexamethasone, 50 mg of diphenhydramine, 20 mg of famotidine, and 8 mg of ondansetron to decrease the risk of an allergic reaction. The treatment proceeded using the modified oral metronidazole desensitization protocol (Table 1). A baseline reading of vitals was taken before metronidazole administration. One ICU nurse and medical student monitored the patient for signs and symptoms of reaction and recorded vital signs for each hour during treatment and for an additional hour after the final dose (Table 2). No adverse reactions during the course of treatment were reported. A prescription was given for the patient’s sexual partner(s) as standard protocol for the treatment of trichomoniasis. The patient was contacted after discharge by the clinical team and reported resolution of her presenting symptoms.

**Table 1:** Metronidazole desensitization dilution protocol.

Solution 1: Using metronidazole 200 mg/5 ml oral suspension draw out 0.1 ml (4 mg) dilute further in 10 ml sterile water (0.4 mg/ml). Take 0.4 mg/ml dilute it further in 10 ml to give (0.4 mg/ml). Take 0.04 mg/ml dilute it further in 10 ml to give (0.004 mg/ml). Take 0.004 mg/ml dilute it further to give 0.0004 mg/ml				
Time interval (minutes)	Dosage	Volume	Dose verified by	Reaction
0	0.00025 mg	6.25 ml		
30	0.025 mg	6.25 ml		
60	0.25 mg	6.25 ml		
90	2.5 mg	6.25 ml		
Solution 2: Using metronidazole 200 mg/5 ml oral suspension. Draw out 1 ml and further dilute to 10 ml with sterile water new concentration (4 mg/ml)				
Time interval (minutes)	Dosage	Volume	Dose verified by	Reaction
120	5 mg	1.25 ml		
150	10 mg	2.5 ml		
180	25 mg	6.25 ml		
210	50 mg	12.5 ml		
240	100 mg	25 ml		
Solution 3: Using metronidazole 200 mg/5 ml undiluted (do not dilute)				
Time interval (minutes)	Dosage	Volume	Dose verified by	Reaction
270	250 mg	6.25 ml		
300	500 mg	12.5 ml		
330	1000 mg	25 ml		

**Table 2:** Vital signs recorded for case 1. SBP: systolic blood pressure, DBP: diastolic blood pressure, HR: heart rate, and RR: respiratory rate.

Time	Dosage	SBP	DBP	HR	RR
08:25	Baseline	121	84	90	28
09:30	Dose 1, 0.0025 mg	111	81	80	20
10:00	Dose 2, 0.025 mg	109	74	76	17
11:00	Dose 4, 2.5 mg	109	73	58	16
12:00	Dose 6, 10 mg	106	62	56	20
13:00	Dose 8, 50 mg	101	60	70	24
14:00	Dose 10, 250 mg	121	77	70	18
15:00	Dose 12, 1000 mg	119	65	90	24
16:00	Post-treatment	132	68	81	24

Case 2

A 53-year-old black female presented via a telehealth appointment with complaints of vaginal itching, burning of the perineal region, burning upon micturition, and foamy green vaginal discharge. The patient presented initially about two years prior to this appointment for an ongoing history of bacterial vaginosis and persistent trichomoniasis. After initial treatment at that time, the patient experienced an anaphylactic reaction within 30 minutes of receiving oral metronidazole, which was characterized by scalp and skin burning, tongue and throat edema, with respiratory distress and hypotension, for which she required emergency treatment. However, after being discharged from emergency services, the patient was lost to follow-up. Two-years later, the patient presented again to her primary physician with complaints that her vaginal symptoms persisted. Upon referral to the infectious diseases' specialist, the patient was counseled on the treatments for trichomoniasis, with the conclusion that other antimicrobials in the 5-nitroimidazole class (including tinidazole) posed a serious risk given her history of anaphylactic reaction. The specialist discussed both furazolidone and hamycin as possible treatments as their efficacy had been remotely described for trichomoniasis. However, these antimicrobial medications were not readily obtainable by the facilities. It was discussed with the patient that the most efficacious treatment would be metronidazole given in smaller doses over a long period of time while being monitored in the ICU. A vaginal culture revealed trace white blood cells, absent yeast, and confirmed the presence of *Trichomonas vaginalis*. After consent was obtained, the patient was scheduled for metronidazole desensitization with continuous monitoring in the ICU.

The patient was pretreated with 20 mg of dexamethasone, 50 mg of diphenhydramine, 20 mg of famotidine, and 8 mg of ondansetron intravenously. Vital signs were recorded at baseline and for each hour during treatment (Table 3). No adverse reactions were observed or reported by the patient. Due to abnormal vital signs observed at 17:00 hours, the clinical team monitored the patient in the ICU for an additional period until vitals stabilized. Prior to discharge, a prescription was given for the patient’s sexual partner(s) as standard protocol. The patient was contacted post-discharge, to which she reported resolution of symptoms.

**Table 3:** Vital signs recorded for case 2. SBP: systolic blood pressure, DBP: diastolic blood pressure, HR: heart rate, and RR: respiratory rate.

Time	Dosage	SBP	DBP	HR	RR
09:15	Baseline	135	69	62	28
11:00	Dose 1, 0.0025 mg	123	74	55	40
12:00	Dose 3, 0.25 mg	145	74	57	16
13:00	Dose 5, 5 mg	128	68	59	22
14:00	Dose 7, 25 mg	145	84	54	34
15:00	Dose 9, 100 mg	125	75	50	16
16:00	Dose 11, 500 mg	145	73	55	18
17:00	Post-treatment	165	70	60	25
18:20	Post-treatment	146	65	55	19

## Discussion

To our knowledge, two desensitization protocols for metronidazole administration are published in the literature. The clinical team chose to follow the protocol published by Gendelman et al. [5] as opposed to that of Kurohara et al. [8]. The rationale for this decision was that the latter study did not document time intervals between metronidazole doses (Table 4). In our clinical opinion, we believed that the former study's gradual dose escalation would decrease the chance of severe systemic reaction.

**Table 4:** Protocols for oral desensitization to metronidazole in the literature. *Thirty-minute intervals between each dose. #No specified time interval between each dose. §Stop at this dose if metronidazole is being prescribed at 500 mg orally twice per day for seven days. ¶ Continue to this dose if metronidazole is being prescribed at two grams orally in a single dose.

Modified protocol*, Gendelman et al. (2014)	Protocol#, Kurohara et al. (1991)
Dose 1, 0.0025 mg	Dose 1, 0.0025 mg
Dose 2, 0.025 mg	Dose 2, 0.025 mg
Dose 3, 0.25 mg	Dose 3, 0.25 mg
Dose 4, 2.5 mg	Dose 4, 2.5 mg
Dose 5, 5 mg	
Dose 6, 10 mg	
Dose 7, 25 mg	Dose 5, 25 mg
Dose 8, 50 mg	
Dose 9, 100 mg	
Dose 10, 250 mg	Dose 6, 250 mg
Dose 11, 500 mg§	Dose 7, 750 mg
Dose 12, 1000 mg¶	Dose 8, 1000 mg

Skin prick and intradermal testing were not administered to our patients as was done by other studies to classify the type of reaction to metronidazole, as cutaneous testing for metronidazole hypersensitivity was not available at our institution [5]. Two studies reported that the validity of skin testing for metronidazole in determining the etiology of a reaction is not validated, and the negative predictive value is unknown [5,13]. In the Gendelman study, one patient was given skin and intradermal testing for metronidazole, which were both negative. However, during the desensitization procedure, the patient developed lip tingling and pruritus 15 minutes after the 10 mg dose [5]. More studies may need to be carried out to determine with confidence the validity and predictive values of these tests.

The patients in our case report did not present with any obvious signs or symptoms during the desensitization that would have indicated a life-threatening IgE-mediated hypersensitivity reaction. Two sets of vital signs were recorded for both patients during the desensitization, one from the telemetry machine and the other manually by the nurse. The telemetry machine recorded tachypnea in Case 1, with respiratory rates varying between 20 and 43 breaths per minute (not shown). The nurse’s reported vital signs were more similar to the patient’s baseline, and hence we have only reported the nurse’s manually documented vitals. With the absence of wheezing, dyspnea, use of accessory muscles or intercostal retractions that would indicate respiratory distress, we concluded that there may be some inaccuracies in the telemetry machine recording. In Case 2, a rise in systolic pressure was observed as well as respiratory rates varying between 16 and 40 breaths per minute. We are uncertain of the etiology of these abnormal vital signs, and we cannot say with confidence if they were a direct consequence of the metronidazole doses, patient anxiety, or other etiologies. The patient in Case 2 was not observed to be in distress or unstable despite these anomalies.

## Conclusions

In patients with a reported or suspected history of IgE-mediated hypersensitivity reactions to antimicrobials, physicians must educate their patients on the risks of untreated STIs as well as the potential adverse outcomes of treatment. In cases such as the ones described in this report, where *Trichomonas vaginalis* is only sensitive to the 5-nitroimidazole class and other pharmacologic agents are not readily available, desensitization should be considered in order to prevent the progression of the disease and increased risk for other STIs and transmission to others. As within this desensitization protocol, patients with a known or expected hypersensitivity reaction should be monitored continuously in an acute setting such as an ICU in case a severe reaction ensues. As mentioned in our limitations, we are unsure of the etiologic cause of the abnormal vital signs recorded during the procedure and whether they were the result of a hypersensitivity reaction or other causes. The success of this metronidazole desensitization protocol may offer a safe and efficacious alternative treatment and allow physicians to feel confident in treating patients with hypersensitivity reactions to metronidazole with a clinical diagnosis of trichomoniasis.
